# A Study on the Clinical Phenotypes and Genetic Analysis of *ENG* Variants in Four Hereditary Hemorrhagic Telangiectasia Type 1 Families

**DOI:** 10.1155/humu/8307860

**Published:** 2026-08-21

**Authors:** Yujing Gong, Tingmin Zhou, Xinru Fu, Yiyi Jiang, Danping Wang, Chuangjie Gu, Ruiting Wu, Dan Wang, Chang Yu

**Affiliations:** ^1^ Special Examination Department, The Affiliated Women and Children′s Hospital of Jiaxing University, Jiaxing, Zhejiang, China; ^2^ Department of Pediatrics, The First Affiliated Hospital of Wenzhou Medical University, Wenzhou, Zhejiang, China, wzhospital.cn; ^3^ Department of Pediatrics, Taizhou Hospital of Zhejiang Province Affiliated to Wenzhou Medical University, Linhai, Zhejiang, China, wmu.edu.cn; ^4^ Department of Radiography, The First Affiliated Hospital of Wenzhou Medical University, Wenzhou, Zhejiang, China, wzhospital.cn; ^5^ Interventional Radiology Department, The First Affiliated Hospital of Wenzhou Medical University, Wenzhou, Zhejiang, China, wzhospital.cn

**Keywords:** *ENG* gene, functional analysis, hereditary hemorrhagic telangiectasia, pulmonary arteriovenous malformation

## Abstract

**Background:**

Hereditary hemorrhagic telangiectasia (HHT) is an autosomal dominant vascular disorder primarily caused by pathogenic variants in the *ENG* gene, leading to clinical manifestations including pulmonary arteriovenous malformation (PAVM), recurrent spontaneous nosebleeds, and other related symptoms. This study is aimed at investigating the clinical manifestations of members in four HHT1 families with PAVMs and analyze novel *ENG* variants.

**Methods:**

Clinical evaluations, whole exome sequencing (WES), and Sanger sequencing were performed on the probands and their family members from Families 1 to 4. Bioinformatics programs were utilized to assess the pathogenicity of the candidate variants. Additionally, an in vitro minigene assay was conducted to examine the impact of the variant in Family 2 on RNA splicing, and Western blot was employed to validate the impact of the variant on protein expression and modification.

**Results:**

Four *ENG* variants were identified: c.613del (exon 5), c.1428 + 2 T > C (intron 11), c.1498dup (exon 12), and c.322del (exon 3). The splice‐site variant c.1428 + 2 T > C, which has been reported as pathogenic in ClinVar, leads to aberrant *ENG* mRNA splicing with exon 11 skipping, resulting in a shorter protein (p.Lys438_Gln476del) with impaired glycosylation.

**Conclusion:**

The splice‐site variant c.1428 + 2 T > C caused aberrant ENG mRNA splicing, leading to exon 11 skipping and producing a shorter protein with abnormal glycosylation (p.Lys438_Gln476del). The variants identified in Families 1, 3, and 4 (c.613delC, c.1498dupC, and c.322delG) are all frameshift variants that lead to premature termination of translation. This study expands the spectrum of *ENG* variants and provides insights into the pathogenesis of HHT1. These findings offer guidance for the early diagnosis for families affected by HHT1.

## 1. Introduction

Pulmonary arteriovenous malformation (PAVM) is characterized by abnormally dilated blood vessels where arterial blood, not oxygenated by capillaries, directly enters the veins, creating a right‐to‐left shunt between the pulmonary artery and venous circulation [1]. The clinical manifestations of children diagnosed with PAVM are diverse, mainly including symptoms and signs such as cyanosis, difficulty breathing, clubbing of fingers, dizziness, fatigue, and decreased blood oxygen saturation [2]. The diagnosis of PAVM primarily relies on imaging examinations. Chest computed tomography (CT) scanner examination, computed tomography angiography (CTA), and digital subtraction angiography (DSA) are the main tools. CT scanning is considered the preferred imaging modality for diagnosis and plays a crucial role in diagnosis, treatment planning, and follow‐up [3]. With the advancement of catheter embolization techniques, percutaneous embolization has become the preferred treatment method for PAVM [4, 5].

The most common cause of PAVMs is hereditary hemorrhagic telangiectasia (HHT), a vascular malformation disease inherited in an autosomal dominant pattern [6, 7]. The *ENG* gene is located at the 9q34.11 region [8]. Variations in the *ENG* gene (encoding endoglin [ENG]) and the *ACVRL1* gene (encoding the activin receptor‐like kinase 1 [ALK1]) cause HHT1 (OMIM #187300) and HHT2 (OMIM #600376), respectively [8, 9]. The *ENG* and *ACVRL1* genes encode proteins of the TGF‐*β* receptor. ENG is a transmembrane glycoprotein found on endothelial cells that serves as a coreceptor for various ligands of the transforming growth factor‐beta (TGF‐*β*) family. The TGF‐*β* signaling pathway plays a crucial role in regulating processes such as cell proliferation, differentiation, adhesion, and migration, ultimately affecting the structure and function of blood vessels [10].

The main clinical manifestations of HHT include the following four aspects: (1) recurrent and refractory epistaxis (nosebleeds); (2) cutaneous and mucosal telangiectasia; (3) visceral arteriovenous malformations (AVMs), with the most commonly affected organs being the lungs, liver, gastrointestinal tract, and brain; and (4) family history. The currently accepted clinical diagnostic criteria for HHT are the Curaçao Criteria established in 1999 [11]: A definite diagnosis is made if three or more of the above four criteria are met; a suspected diagnosis is considered if only two of the four criteria are met; if only one of the above criteria is met, HHT is generally not diagnosed.

In this study, we reported four families with patients diagnosed with PAVMs, whose clinical manifestations are consistent with HHT. Through whole exome sequencing (WES), variations in the *ENG* gene were identified in all four families. This includes previously unreported variants (c.1428 + 2 T > C, c.1498dup, and c.322del), thereby expanding the variation spectrum of the *ENG* gene. In addition, we performed in silico analysis to explore the possible molecular pathogenesis of HHT in these families.

## 2. Materials and Methods

### 2.1. Participants

The probands and families were enrolled at the First Affiliated Hospital of Wenzhou Medical University. Relevant clinical records were collected, including physical examination, blood routine test, ultrasound examination, chest CT scan, and pulmonary angiography. Written consent was obtained from the patients′ parents prior to the commencement of the study, and approval was obtained from the Ethics Committee of the First Affiliated Hospital of Wenzhou Medical University.

### 2.2. Trio WES and Sanger Sequencing

The genomic DNA extracted from 1 to 2 mL of blood samples obtained from the probands and their family members served as the material for the assay. The process began with extracting 1 *μ*g of genomic DNA from 200 *μ*L of peripheral blood using a Qiagen DNA Blood Midi/Mini kit (Qiagen GmbH, Hilden, Germany). Fragmentation enzymes then broke down 50 ng of DNA into approximately 200‐bp fragments, which were subsequently end repaired and had a single A base added to the 3 ^′^ end. Barcoded sequencing adaptors were then ligated to the DNA fragments, with those around 320 bp being collected using XP beads. Following PCR amplification, the DNA fragments underwent hybridization and capture using Berry′s NanoWES Human Exome V1.0 kit (Berry Genomics, Beijing, China). The hybrid products were eluted, collected, and subjected to further PCR amplification and purification. Subsequently, the libraries were quantified using qPCR, and their size distribution was determined using an Agilent Bioanalyzer 2100 (Agilent Technologies, Santa Clara, California, United States). Finally, the genomic DNA of the family was sequenced using the Novaseq6000 platform (Illumina, San Diego, United States) in 150‐bp pair‐end mode, with raw image files processed using CASAVA v1.82 for base calling and data generation. The sequencing reads were aligned to the human reference genome (hg38/GRCh38) using the Burrows‐Wheeler Aligner tool, and PCR duplicates were removed with Picard v1.57 (http://picard.sourceforge.net/). Variant calling was performed using the Verita Trekker Variants Detection System by Berry Genomics and the third‐party software GATK (https://software.broadinstitute.org/gatk/). Annotation and interpretation of variants were carried out using ANNOVAR (https://annovar.openbioinformatics.org/en/latest/) and the Enliven Variants Annotation Interpretation System authorized by Berry Genomics. Annotation databases primarily included human population databases (gnomAD and the 1000 Genome Project), in silico prediction algorithms (SIFT and FATHMM), and disease/phenotype databases (OMIM and ClinVar). This is used to determine which category the variant is judged to be in, and further reference is provided for subsequent research. The suspected mutations were subsequently verified using Sanger sequencing in the studied families (Table 1). Sanger sequencing was performed using an ABI 3730xl DNA Analyzer (Applied Biosystems, Carlsbad, California, United States).

**Table 1:** The primers for Sanger sequencing of Families 1–4.

Family	Primer sequence
Family 1	F: CTATCTTTGGCTGTGGGTGAGGG
	R: TGGTCTCTCGGGGTGGGGA
Family 2	F: TCAGGCAACTCCACAGGGCCAT
	R: CAGTGCCCCAGACACAGCAGTCCC
Family 3	F: tgtaaaacgacggccagtGGAAGCTCCCACTTGAAGC
	R: caggaaacagctatgaccGACTCAGGGGTGGGAACTC
Family 4	F: ACTGTGTGTGTCTCTCCTCTGTGT
	R: CCCTGGTGAATAATGTCAAGATG

### 2.3. In Silico Assay

To analyze the possible impact of the mutation, the Rare Disease Data Center (RDDC) database (https://rddc.tsinghua-gd.org/) was utilized. SpliceAI (https://spliceailookup.broadinstitute.org/#) was used for the mutation evaluation. The prediction procedure adhered to standard methodologies.

### 2.4. Minigene Constructs and Mutagenesis

To examine the target gene regions covering *ENG* exons 10–12 and introns 10–11, which were amplified from the gDNA of the proband from Family 2, an in vitro minigene assay was performed. The wild‐type (WT) *ENG* gene fragment was acquired through nested polymerase chain reaction (PCR), employing genomic DNA as a template and using the primers 34187‐F/36503‐R and 34424‐F/36503‐R (Table 2). Three pairs of primers (pcDNA3.1‐*ENG*‐BamHI‐F/*ENG*‐mut‐R, *ENG*‐mut‐F/pcDNA3.1‐*ENG*‐XhoI‐R, and pcDNA3.1‐*ENG*‐BamHI‐F/pcDNA3.1‐*ENG*‐XhoI‐R) were devised to amplify the heterozygous c.1428 + 2 T > C mutation site from the nested PCR product through seamless cloning (Vazyme Biotech Co. Ltd., Nanjing, China). Subsequently, the amplified DNA products were recombined and inserted into two restriction sites (BamHI/XhoI) of the pcDNA3.1 vector (Hitrobio Biotechnology Co. Ltd., Beijing, China). In addition, the recombinant plasmids pcDNA3.1‐*ENG*‐t (WT) and pcDNA3.1‐*ENG*‐mut (c.1428 + 2 T > C) were confirmed via Sanger sequencing.

**Table 2:** Primer sequences for *ENG* gene variant analysis in Family 2 and pcDNA3.1 vector.

Primer name	Primer sequence (5 ^′^–3 ^′^)
34187‐F	atgaagagggagcagggcag
34424‐F	ATGCAGGTGTCAGCAAGTAT
36503‐R	gacgggtaagtgaaaggatg
36754‐R	aatcctggaggcctgtctgt
pcDNA3.1‐ENG‐BamHI‐F	GCTCGGATCCATGGCGGTGGTCAATATCCTGTC
ENG‐mut‐F	AGAGCTTTGTGCAGgCacctggcatgcctgt
ENG‐mut‐R	acaggcatgccaggtGcCTGCACAAAGCTCT
pcDNA3.1‐ENG‐XhoI‐R	TAGACTCGAGCTGGTCTTGAGACCCGGTCT
pcMINI‐C‐ENG‐BamHI‐F	GCTCGGATCCggcagatcacgaggtgaaga
pcMINI‐C‐ENG‐XhoI‐R	TAGACTCGAGCTGGTCTTGAGACCCGGTCT
pcDNA3.1‐F	CTAGAGAACCCACTGCTTAC
pcDNA3.1‐R	TAGACTCGAGCTGGTCTTGAGACCCGGTCT
pcMINI‐C‐F	ACTTAAGCTTatgagtgggctttggggtggccggtt
pcMINI‐C‐R	TAGAAGGCACAGTCGAGG

To corroborate the conclusions derived from this study, the assay was replicated using the same experimental protocol, using a pcMINI‐N vector that includes the universal Exon A‐intron A‐MCS sequence.

### 2.5. Cell Culture and Transfection

Human embryonic kidney (HEK293T) and HeLa cell lines (Cell Resource Center of the Chinese Academy of Medical Science, Beijing, China) were cultured at 37°C in a humidified 5% CO_2_ environment using Dulbecco′s modified Eagle medium supplemented with 10% fetal bovine serum and 1% penicillin–streptomycin. HEK293T and HeLa cells were transfected using the Lipo3000 transfection reagent (GlpBio Technology Inc., Montclair, California, United States). The transfected cells were incubated for 48 h before RNA extraction.

### 2.6. RNA Extraction, PCR, and Sequencing

Total RNA was isolated from HEK293T and HeLa cells transfected with the minigene plasmids using the GenElute Mammalian Total RNA Kit (Sigma‐Aldrich) as per the manufacturer′s instructions. DNA was subsequently removed from the RNA preparation through digestion with DNase I (Qiagen, Valencia, California, United States) on the columns. The purified total RNA was then reverse transcribed into cDNA, and a primer set was employed for the retro‐PCR targeting pcDNA3.1‐F/pcDNA3.1‐R (Table 2). Subsequently, the cDNA products were analyzed using 1% agarose gel electrophoresis and confirmed by Sanger sequencing. The lengths of the WT cDNA products were identified as 533 bp in the pcDNA3.1‐*ENG* vector and 635 bp in the pcMINI‐N‐*ENG* vector.

### 2.7. Western Blot Analysis

A plasmid expressing human *ENG* and another plasmid (p.Lys438_Gln476del‐ENG‐FLAG) containing the *ENG* bearing the same variant as the proband of Family 2 were obtained from Miaoling Bioscience (Wuhan, China). All the plasmids were confirmed by sequencing (Tsingke Biotechnology Co. Ltd, Beijing, China). Primer sequences for generating p.Lys438_Gln476del were as follows: D1 (forward): GTAGGCGTGTACGGTGGGAG and DR (reverse): gctgtttacactgaggacc. Equal amounts of the two types of plasmids were subsequently transiently transfected into HEK293T cells using Lipo2000 transfection reagent (GlpBio Technology Inc., Montclair, California, United States). After 48 h, cells were harvested using ice‐cold PBS and lysed in RIPA buffer with protease inhibitors (Tris‐HCl, 50 mM; Triton X‐100, 1%; deoxycholate, 1%; NaCl, 150 mM; and SDS, 10%). The protein concentration was determined using the Pierce BCA Protein Assay Kit (Thermo Fisher Scientific, Waltham, Massachusetts, United States). The cell lysates were separated on 10% Tris–glycine sodium dodecyl sulfate–polyacrylamide gel electrophoresis (SDS‐PAGE) gels. Subsequently, proteins were transferred to PVDF membranes. Monoclonal ANTI‐FLAG M2, Clone M2 (1:1000; Sigma‐Aldrich, St. Louis, Missouri, United States) was used for WB analysis according to standard protocols. The blot was incubated with goat antimouse IgG HRP secondary antibody (Biosharp, Hefei, Anhui, China), diluted 1:10000, at room temperature for 1 h. Afterwards, the blot was washed with tris‐buffered saline with Tween 20 (TBST), and finally, film exposure, development, and image capture were performed.

## 3. Results

### 3.1. Clinical Data

#### 3.1.1. Family 1

**Figure 1:**
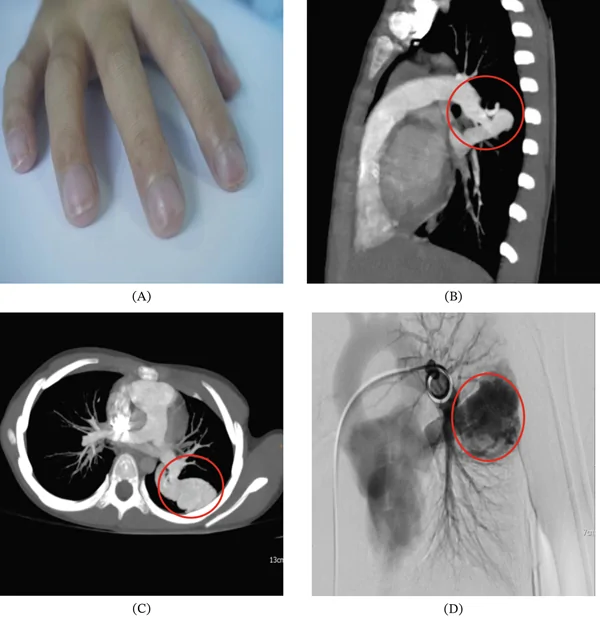
Imaging of the proband in Family 1. (A) Clubbing of fingers, slight cyanosis at the fingertip. (B) Oblique sagittal MIP shows supplying pulmonary artery and draining vein. (C) Axial plane shows tortuous and enlarged vascular shadow in the posterior segment of the left lower lobe. (D) Arterial DSA angiography confirms it as a cystic PAVF.

The proband is a 7‐year‐old girl hospitalized for fatigue. Physical examination revealed that the child had shortness of breath and mild cyanosis at the fingertips (Figure 1A), with the peripheral oxygen saturation ranging from 83% to 85%. There was also significant growth and developmental delay: weight was 18 kg (−1.3 SD, 5th percentile); height was 116 cm (−1.4 SD, 10th percentile). Her complete blood count revealed a hemoglobin of 168 g/L, hematocrit of 0.488 L/L. Contrast‐enhanced pulmonary angiography (CTPA) suggested the presence of PAVMs (Figure 1B,C). The patient underwent successful percutaneous endovascular embolotherapy of the arteriovenous fistula (Figure 1D). Follow‐up at 3 months showed the child was catching up in growth and development: Weight was 18.6 kg (−1.2 SD, 10th percentile); height was 118 cm (−0.8 SD, 25th percentile).

Further history revealed a family history of recurrent nosebleeds in the mother, sister, and brother (Table 3).

**Table 3:** Clinical manifestations.

	Family 1				Family 2		Family 3		Family 4			
	Proband	Mother	Sister	Brother	Proband	Mother	Proband	Mother	Proband	Mother	Aunt	Cousin
Nosebleed	+	+	+	+	+			+				+
Capillary dilation					+							
Pulmonary arteriovenous malformation	+				+	+	+	+			+	
Cerebral arteriovenous malformation					+		+				+	
Hepatic arteriovenous malformation						+						
Dyspnea	+											
Cyanosis	+											
Intracerebral hemorrhage									+			
Cerebral infarction					+						+	
Anemia							+					
Erythrocytosis	+											
Clubbing of fingers	+							+				
Epilepsy									+			
Spontaneous abortion								+				
HOHF												
Growth and development delay	+						+		+			

#### 3.1.2. Family 2

**Figure 2:**
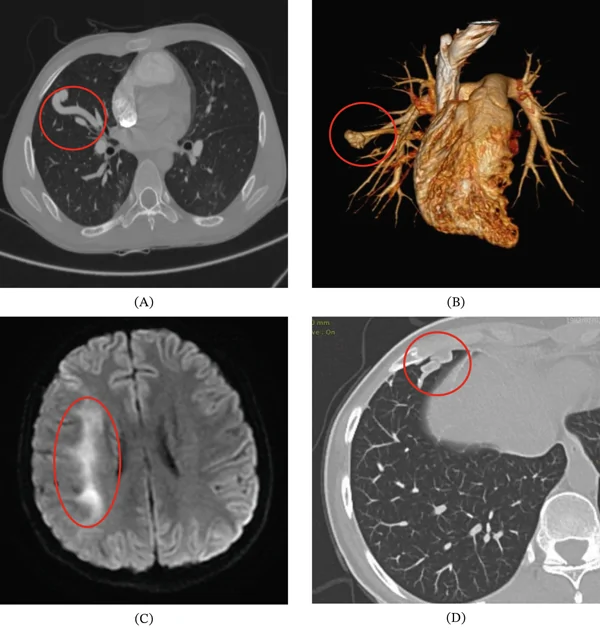
Imaging of Family 2. (A) Proband: Axial plane shows a large ductal shadow and nodular shadow in the right middle lobe of the lung. (B) Proband: VR shows an aneurysm sac, supplying pulmonary artery, and draining vein. (C) Proband: Cranial DWI shows high signal in the right corona radiata. (D) The proband′s mother: Axial plane shows tortuous and enlarged vascular shadow in the right middle lobe of the lung.

The proband is a 13‐year‐old boy who has a history of recurrent spontaneous epistaxis. Nasal endoscopy revealed telangiectases on the left nasal septum. He was admitted for transient syncope accompanied by facial asymmetry. Physical examination on admission revealed left facial palsy (nasolabial flattening with rightward oral commissure deviation), left hemiparesis (upper limb 0/5, lower limb 4–5/5 by MRC scale), left‐sided hypesthesia, and preserved right‐sided strength (5/5). Cranial magnetic resonance imaging (MRI) demonstrated a cerebral infarction in the right hemisphere. Chest CT revealed a large tubular shadow and nodules in the right middle lobe, with smaller vascular dilatations in the right lower lobe. CTPA showed an arteriovenous malformation in the right middle lobe and bilateral lower lobe infections (Figure 2A,B). The patient underwent cerebral angiography and intracranial mechanical thrombectomy, with intraoperative diagnosis of right middle cerebral artery (MCA) occlusion (Figure 2C). Posttreatment, he reported mild weakness during ambulation, with near‐complete recovery of left limb muscle strength. At the 1‐month and 3‐year follow‐ups, he underwent partial percutaneous embolotherapy of the right pulmonary artery.

Further history revealed a family history of PAVM in the mother (Table 3). Chest CT indicated dense linear opacities and abnormal vasculature in the anterior segment of the right upper lobe (Figure 2D). Abdominal ultrasound revealed a hyperechoic mass in the right liver, suggestive of hemangioma, with concomitant calcifications. The mother also underwent percutaneous embolotherapy. The father has no clinical manifestations.

#### 3.1.3. Family 3

**Figure 3:**
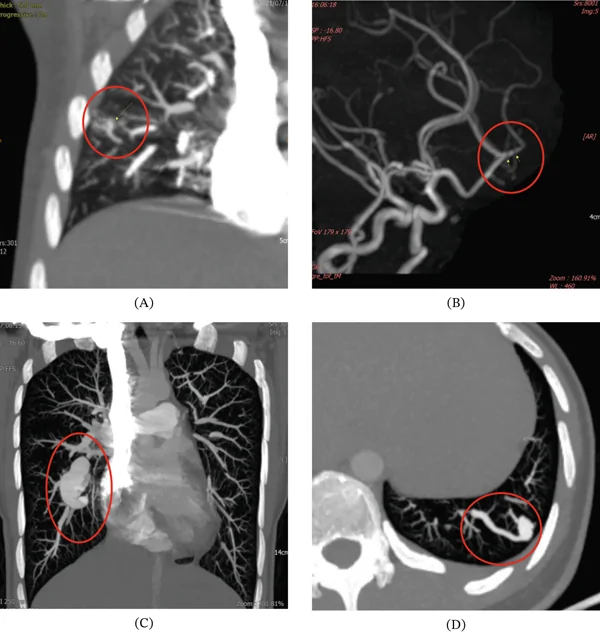
Imaging of Family 3. (A) Proband: Oblique coronal MIP shows suspected arteriovenous malformation in the right middle lobe of the lung. (B) Proband: MRA indicates that the A3–4 segment of the left anterior cerebral artery appears slender, whereas the M2 segment of the right middle cerebral artery shows localized mild stenosis. (C) Proband′s mother: Sagittal plane shows an aneurysm sac in the right lung, with supplying pulmonary artery and draining vein. (D) Proband′s mother: Postpulmonary embolism surgery: MIP shows a dense shadow in part of the arterial branches in the right lower lobe of the lung, with strip‐shaped filling defects in the distal areas.

The proband is a 2.5‐year‐old girl who was admitted for evaluation of growth and developmental delay. At 21 weeks of gestation, fetal ultrasound demonstrated a 1‐week growth discrepancy. She was delivered at the 39th week weighing 2200 g (<−2 SD, small for gestational age infant), accompanied by a thin umbilical cord and small placenta. She achieved gross motor milestones as follows: head control at 4 months, rolling over at 5 months, independent sitting at 8–9 months, crawling at 10 months, and independent walking at 1.5 years. Language development was delayed, with no meaningful vocalizations before 1 year, and only 2–3 word combinations at 2.5 years. There was significant growth and developmental delay: Weight was 10.8 kg (<−2 SD, < 3rd percentile) and height was 87 cm (<−2 SD, < 3rd percentile). CTPA indicated a suspicious local vascular shunt in the right middle lobe with distal inflammatory lesions (Figure 3A). Cerebral magnetic resonance angiography (MRA) revealed that the A3–4 segments of the left anterior cerebral artery appeared slender, with mild focal stenosis in the M2 segment of the right MCA, and a fetal‐type left posterior cerebral artery (Figure 3B). Abdominal ultrasound revealed splenomegaly, whereas cardiac echocardiography showed no abnormalities.

Further history revealed that the mother had experienced two spontaneous abortions and recurrent nosebleeds (Table 3). Physical examination showed finger clubbing. CTPA demonstrated arteriovenous malformations in both lower lung lobes (Figure 3C,D). She also underwent percutaneous embolotherapy. The father has no clinical manifestations.

#### 3.1.4. Family 4

The proband is an 11‐month‐old boy who was delivered vaginally at 37^+6^ weeks. At birth, the infant had a weight of 2470 g (−1 SD). At 34 weeks of gestation, the mother noticed abnormal fetal movements. Prenatal MRI demonstrated extensive subdural hematomas in the left cerebral hemisphere and left posterior fossa, with bilateral cerebral hemispheric encephalomalacia. The infant presented with dysmorphic facial features, followed by a weakness in sucking and necessitating nasal feeding. Subsequent follow‐ups revealed significant developmental delay and cognitive impairment.

**Figure 4:**
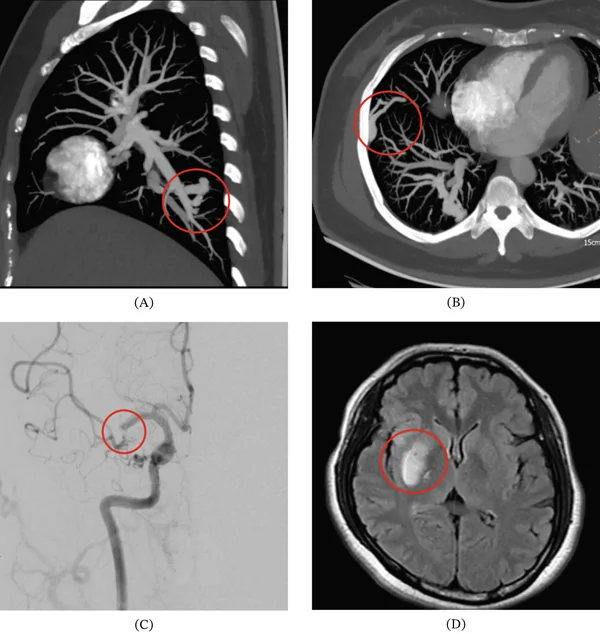
Images of the proband′s aunt in Family 4. (A) Sagittal plane MIP shows communication between the posterior basal segment of the right lower lobe and the right inferior pulmonary vein. (B) Axial plane MIP shows a vascular‐like enhancing nodule in the lateral segment of the right middle lobe. (C) Right intracranial artery DSA shows partial occlusion of the M2 segment of the right middle cerebral artery with formation of abnormal collateral vessels. (D) Head MRI, T2‐FLAIR shows abnormal signal in the right basal ganglia region.

The proband′s aunt was hospitalized for left‐sided limb weakness. CTPA indicated arteriovenous malformations in the lateral segment of the right middle lobe and the basal segment of the right lower lobe, with filling defects in the pulmonary veins and branches of the right middle lobe, indicating thrombosis (Figure 4A,B). Cerebral angiography revealed a fetal‐type left posterior cerebral artery and mild hypoplasia of the right MCA (M2–M3 segments) (Figure 4C). Cranial MRI demonstrated an acute infarction in the right fronto‐parieto‐insular periventricular basal ganglia region (Figure 4D). The aunt underwent cerebral angiography with intracranial mechanical thrombectomy and embolotherapy of the pulmonary artery.

The maternal grandfather died prematurely due to cerebral infarction (no WES performed). The aunt′s son has recurrent nosebleeds, whereas her daughter shows no clinical manifestations (Table 3). The proband′s father has no clinical manifestations.

### 3.2. Variant Detection

#### 3.2.1. Family 1

**Figure 5:**
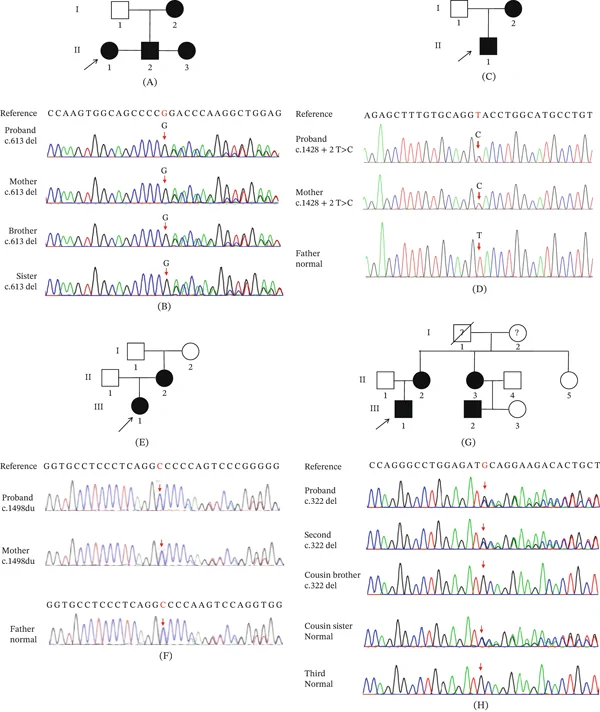
Pedigree and the sequencing result of the studied families. □ represents the normal male; ○ represents the normal female; ■ represents the affected male; ● represents the affected female. The black arrow indicates the proband. The red arrow symbol indicates the mutation site. (A, B) Family 1. (C, D) Family 2. (E, F) Family 3. (G, H) Family 4.

A heterozygous deletion variant in exon 5 of *ENG* (c.613del; NM_000118.3) was identified in the proband (II‐1; Figure 5A) by PCR‐based Sanger sequencing (Figure 5B). DNA analysis revealed that the mother, brother, and sister (I‐2, II‐2, and II‐3) all carry the heterozygous variant.

#### 3.2.2. Family 2

A heterozygous point variant (c.1428 + 2 T > C; NM_000118) in exon 11 of *ENG* was identified in the proband (II‐1; Figure 5C) by PCR‐based Sanger sequencing (Figure 5D). DNA analysis confirmed that the mother (I‐2) also carries the heterozygous variant.

#### 3.2.3. Family 3

A heterozygous duplication variant (c.1498dup; NM_001114753.2) in exon 12 of *ENG* was identified in the proband (III‐1; Figure 5E) by PCR‐based Sanger sequencing (Figure 5F). DNA analysis revealed that the mother (II‐2) carries the heterozygous variant, indicating that the variant is a de novo variant originating from the mother (II‐2).

#### 3.2.4. Family 4

A heterozygous deletion variant (c.322del; NM_000118.4) in exon 3 of *ENG* was identified in the proband (III‐1; Figure 5G) by PCR‐based Sanger sequencing (Figure 5H). DNA analysis showed that the mother, aunt, and cousin (II‐2, II‐3, and III‐2) all carry the heterozygous variant.

### 3.3. In Silico Assay

**Figure 6:**
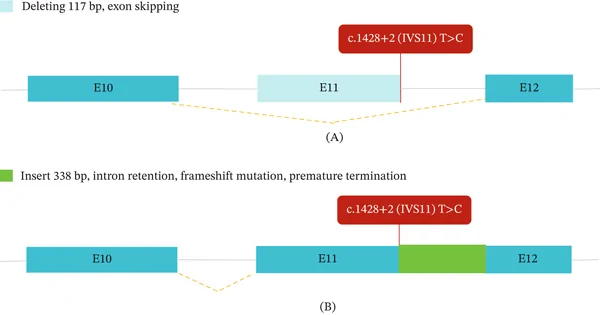
Family 2′s results of in silico assay. (A, B) Patterns of RNA splicing prediction. (A) Deleting 117 bp, exon skipping. (B) Insert 338 bp, frameshift mutation, and premature termination.

In the GnomAD database, the *ENG* variants (c.1428 + 2 T > C, c.1498dup, and c.322del) are not included, indicating that these are novel variants (Table 4). We conducted functional prediction for the *ENG* variants by utilizing the RDDC platform. The assessment revealed the *ENG* variant (c.1428 + 2 T > C) may affect mRNA splicing (Figure 6A,B). For c.1428 + 2 T > C, the splice AI algorithm returned a high value (*Δ* Score = 0.99). This value, which was above the high precision threshold (*Δ* Score ≥ 0.8), was used to detect higher sensitivity of splice change variants (Table 5).

**Table 4:** Variations of *ENG* previously reported in gnomAD database.

Family	Variant ID	HGVS Consequence	VEP Annotation
Family 1 (c.613del: p.Arg205Glyfs∗17)	9‐127825770‐C‐T	p.Arg205Arg	Synonymous
	9‐127825770‐C‐A	p.Arg205Leu	Missense
	9‐127825770‐C‐T	p.Arg205Gln	Missense
	9‐127825770‐C‐C	p.Arg205Gly	Missense
	9‐127825770‐C‐A	p.Arg205Trg	Missense
	9‐127825770‐C‐T	p.Arg205Arg	Synonymous

Family 2 (c.1428 + 2 T > C)	9‐127818707‐G‐A	c.1428 + 9C > T	Intron
	9‐127818709‐G‐T	c.1428 + 7G > A	Splice region
	9‐127818711‐G‐C	c.1428 + 5C > G	Intron
	9‐127818711‐G‐T	c.1428 + 5C > A	Intron
	9‐127818718‐G‐T	p.Gln476Lys	Missense

Family 3 (c.1498dup: p.Glu500Glyfs∗28)	9‐127818305‐C‐G	p.Gly501Arg	Missense
	9‐127818305‐C‐T	p.Glu500Glu	Synonymous
	9‐127818305‐C‐G	p.Glu500Gln	Missense
	9‐127818305‐C‐T	p.Glu500Lys	Missense
	9‐127818305‐A‐G	p.Pro499Pro	Synonymous

Family 4 (c.322del: p.His108Ilefs∗55)	9‐127829722‐G‐A	p.Leu109Phe	Missense
	9‐127829724‐T‐C	p.His108Arg	Missense
	9‐127829725‐G‐A	p.His108Tyr	Missense
	9‐127829725‐G‐T	p.His108Asn	Missense
	9‐127829726‐C‐A	p.Leu107Leu	Synonymous

**Table 5:** Splicing mutation evaluation by SpliceAI.

Acceptor loss	Donor loss	Acceptor gain	Donor gain
0.42	0.99	0.00	0.16
118 bp	2 bp	43 bp	−205 bp

### 3.4. Minigene Assay

**Figure 7:**
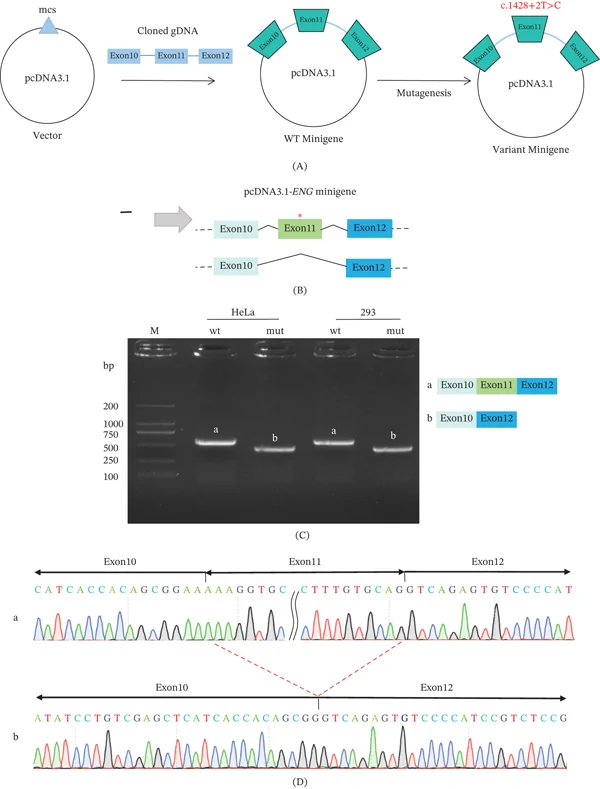
Functional analysis of the effect of the *ENG* c.1428 + 2 T > C variant on mRNA splicing. (A, B) Construction strategy of pcDNA3.1 minigene vector, ∗ representative of the variant site. (C) Gel electrophoresis of reverse transcription polymerase chain reaction products displayed a single band a (estimated 533 bp) from the wild type (wt) and a smaller band b (estimated 416 bp) in the mutant (mut) type. (D) Illustration of the sequencing of band a (wild type in HEK293T and HeLa cells) and band b (variant c.1428 + 2 T > C in HEK293T and HeLa cells) products lead to a shorter transcript with deletion of exon 11 including 117 bp.

In vitro transcriptional assays were performed to analyze the effect of intron donor locus variation on *ENG* mRNA splicing. The pcDNA3.1‐*ENG* small gene was 1541 bp long and covered DNA regions, including Exon10 (3 bp), Intron10 (789 bp), Exon11 (117 bp), Intron 11 (338 bp), and Exon12 (25 bp, Figure 7A,B). A 533‐bp complete reverse transcription PCR product, which includes a 119‐bp segment of plasmid DNA and a 414‐bp segment of the target gene, was observed in HEK293T and HeLa cells after transfection with the pcDNA3.1‐*ENG* minigene construct (Figure 7C). Sanger sequencing verified the 414‐bp fragment as normal *ENG* mRNA with exons 10–12 spliced and introns 10–11 excluded (Figure 7D). The pcDNA3.1‐*ENG*‐mut minigene containing the c.1428 + 2 T > C variant was transfected into the HeLa and HEK293T cell lines. Conversely, WT *ENG* mRNA was absent in the mutant (MUT) swim channel, a smaller band (416 bp, Figure 7C). In the MUT lanes, there was an observation of an *ENG* aberrantly spliced transcript manifesting as a shorter band of 416 bp, encompassing solely exons 10 and 12, with exon 11 completely skipped. Figure 7D displays the Sanger DNA chromatogram of this misspliced *ENG* transcript. The outcomes obtained from the experiment performed using the pcMINI‐C vector aligned with prior findings, demonstrating the skipping of exon 11. Analysis of the complete sequence of the improperly spliced transcript, as determined by small gene analysis, indicated that this variant caused complete skipping of exon 11 without altering the subsequent reading frame. Due to the loss of 117 bp of nucleotides (c.1312_1428del), it resulted in an internal deletion of 39 amino acids (p.[Lys438_Gln476del]) in the protein, potentially leading to a shorter protein. These findings align with predictions from in silico assays, and the RNA splicing pattern corresponds to the outlined splicing pattern I (Figure 6A).

### 3.5. Western Blot Analysis

**Figure 8:**
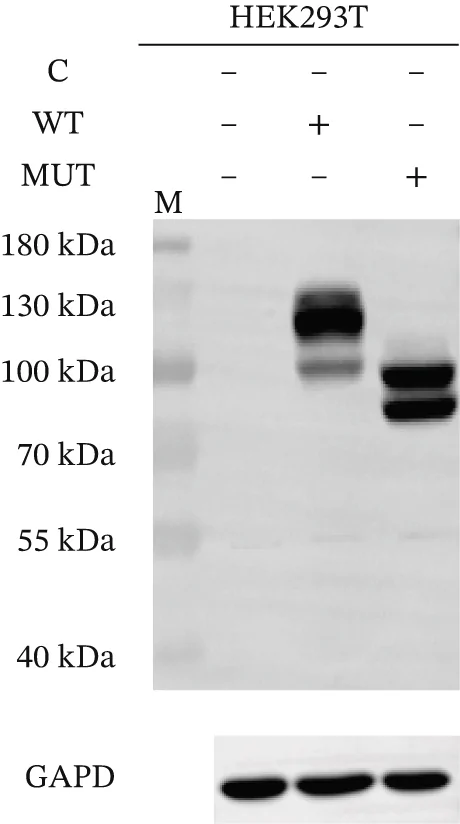
Western blot results on total proteins extracted from transfected 293T cells with the pLV3‐FLAG vector and the p.Lys438_Gln476del construct. Two bands of different molecular weights are observed for ENG proteins likely corresponding to more glycosylated (upper band) and less/nonglycosylated (lower band) ENG monomers. GAPDH corresponds to the used antibody for the reference proteins. kDa, kilodalton; M, protein ladder; WT, wild type; CT, negative control corresponding to pLV3‐empty vector.

Western blot results revealed that the MUT protein exhibited a shorter form (smaller molecular weight) compared with the WT (Figure 8). Both WT and MUT lanes showed two bands: the upper band (glycosylated form) and the lower band (less/nonglycosylated form). Bioinformatics analysis using NetNGlyc predicted that Asn444 (WT sequence: Asn444‐Met445‐Asp446) and Asn465 (WT sequence: Asn465‐Thr466‐Ile467), located within the Lys438‐Gln476 region, are potential glycosylation sites. The minigene assay demonstrated that the c.1428 + 2 T > C mutation disrupted normal splicing, leading to exon 11 skipping (117‐bp deletion) and resulting in the p.Lys438_Gln476del alteration at the protein level. Therefore, the two predicted glycosylation sites at Asn444 and Asn465 are likely deleted in the MUT protein. Western blot results demonstrated that the MUT lane exhibited significantly enhanced accumulation of the lower band compared with the WT lane. This is likely attributable to the loss of N‐glycosylation sites at Asn444 and Asn465 caused by the p.Lys438_Gln476 deletion, which consequently led to elevated expression of the less/nonglycosylated protein. These findings suggest that the *ENG* c.1428 + 2 T > C variant causes exon 11 deletion (117 bp), which corresponds to the p.Lys438_Gln476del mutation and results in a shorter protein (618 aa) and impaired glycosylation.

### 3.6. Three‐Dimensional Structure of the Protein

**Figure 9:**
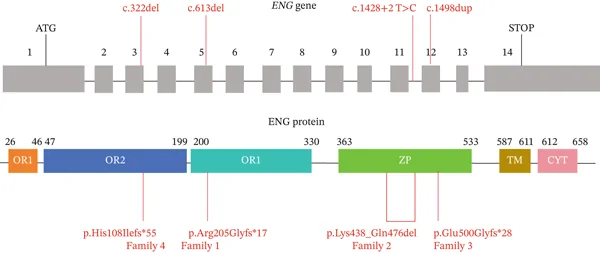
The domain of *ENG* proteins. Structural representation of endoglin. OR: an NH2‐terminal orphan domain. ZP, zona pellucida domain; TM, the transmembrane domain. The cytoplasmic (CYT) domain can be phosphorylated (P) at Ser/Thr/Tyr residues. The scheme is not to scale.

Compared with the WT sequence, the variant protein exhibited an altered amino acid sequence, leading to a shorter protein variant with a deletion of 39 native amino acids p.(Lys438_Gln476del). The variants in the four families differentially affect the functional domains of ENG (Figure 9).

## 4. Discussion

This study conducted a detailed analysis of the clinical phenotypes and genetic characteristics of four families with hereditary hemorrhagic telangiectasia type 1 (HHT1) carrying *ENG* gene variants.

In Families 1–4, patients presented primarily with PAVM as the initial clinical manifestation, with some patients (II‐1 of Family 2 and II‐3 of Family 4) exhibiting severe clinical manifestations and rapid disease progression. PAVM can be classified into primary and secondary types based on the presence or absence of underlying causes, with primary PAVM accounting for approximately 80% of all PAVM cases [12]. About 80%–90% of PAVM patients are eventually diagnosed with HHT [13], whereas at least 50% of HHT patients present with PAVM [14]. Primary PAVM patients with HHT exhibit more pronounced symptoms, faster disease progression, a higher likelihood of multiple PAVMs, and an increased incidence of complications. Patients with PAVMs exhibit diverse clinical manifestations, primarily including cyanosis, fatigue, dyspnea, dizziness, and decreased oxygen saturation. In severe cases, the condition can be life‐threatening. Thrombosis is prone to form within the vascular malformations of PAVMs, and emboli may enter the systemic circulation, leading to conditions such as cerebral ischemia and cerebral infarction (e.g., II‐1 of Family 2 and II‐3 of Family 4) [15]. Anderson et al. [16] suggested in a retrospective study that PAVM might be the only clinical criterion for genetically confirmed HHT. Clinicians and patients are generally aware that PAVMs often occur in a familial setting, most commonly secondary to HHT. For a patient with PAVM and confirmed HHT, each first‐degree relative carries a 50% risk of HHT, and at least 50% of such affected relatives are predicted to have PAVMs. Genetic testing serves as an effective method for diagnosing HHT, regardless of the presence of clinical manifestations, and has become an essential element in the management of patients presenting with one or more PAVMs [1]. Therefore, when encountering patients with one or more PAVMs, clinicians should enhance clinical suspicion and actively pursue HHT genetic testing.

According to the Curaçao Criteria, patients from four families met three diagnostic criteria for HHT, including recurrent spontaneous nosebleeds, mucocutaneous telangiectasia, and a positive family history [11]. Consequently, genetic testing was performed on all four families, revealing *ENG* gene variants in each. Based on the clinical phenotypes and genetic testing results, we confirmed that four families were diagnosed with HHT1. HHT is a rare autosomal dominant vascular disorder with an estimated prevalence of 1 in 5000 to 1 in 8000 [17]. HHT patients exhibit significant phenotypic heterogeneity, with different families displaying varying clinical features, and even within the same family, patients carrying the same pathogenic mutation may present with diverse clinical manifestations. Several studies have indicated that patients with HHT1 are at a greater risk of developing PAVMs and cerebral arteriovenous malformations (CAVMs) compared with those with HHT2 [18, 19]. As demonstrated in the four families reported in this study, clinical phenotypes varied among families, and the severity and progression of the same clinical features also differed (Table 3).

The *ENG* gene is located on human chromosome 9q34.11 and consists of 14 exons [8]. This gene encodes ENG, a homodimeric integral membrane glycoprotein primarily expressed on the surface of vascular endothelial cells. ENG is a crucial component of the TGF‐*β* receptor complex. By associating with the signaling receptors RI and RII, ENG modulates the activity of the TGF‐*β* signaling pathway. ENG mutations may prevent endothelial cells from properly responding to TGF‐*β* signals during angiogenesis, leading to abnormal vascular development, manifested as telangiectasia and arteriovenous malformations [10, 20].

ENG is a Type I transmembrane protein characterized by a large extracellular (EC) domain comprising two functionally distinct regions: an NH2‐terminal orphan domain and a membrane‐proximal zona pellucida (ZP) domain [21]. The orphan domain specifically binds TGF‐*β* superfamily ligands (including activins, TGF‐*β*, and BMPs), consistent with ENG′s function [22]. Meanwhile, the ZP domain facilitates receptor oligomerization, mediates the formation of ENG homodimers/heterodimers, and maintains the stability of the TGF‐*β* receptor complex [23]. The ZP domain also encodes an Arg‐Gly‐Asp (RGD) tripeptide that is a prototypic member of a family of motifs involved in integrin‐based interactions with EC matrix and certain cell surface proteins [24].

In Family 1, the proband carried the c.613del: p.Arg205Glyfs∗17 mutation; in Family 3, the proband carried the c.1498dup: p.Glu500Glyfs∗28 mutation; and in Family 4, the proband carried the c.322del: p.His108Ilefs∗55 mutation. Among pathogenic mutations in the *ENG* gene, small insertions and deletions account for approximately 50%, representing a common type of pathogenic mutation in this gene. In Family 1, the frameshift mutation (c.613del: p.Arg205Glyfs∗17) disrupts the OR1 domain and causes premature protein termination, significantly impairing TGF‐*β* signaling. In Family 4, the frameshift mutation (c.322del: p.His108Ilefs∗55) generates a severely truncated protein (163 aa), with premature protein termination (Figure 9). Phenotypic analysis revealed that Family 4 patients exhibited early‐onset severe symptoms with rapid disease progression, whereas Family 1 patients showed relatively milder clinical manifestations (Table 3). In Family 3, the mutation (c.1498dup: p.Glu500Glyfs∗28) primarily affects the ZP domain. Although ligand‐binding capacity of OR1 and OR3 domains is preserved, impaired oligomerization results in reduced signal transduction efficiency (Figure 9). In Family 3, the clinical phenotype is relatively mild due to retained partial protein function (Table 3). These mutations primarily cause disease by leading to insufficient expression of the *ENG* gene [25, 26]. In this study, the mutations in these three families were all frameshift mutations (c.613del, c.1498dup, and c.322del), resulting in altered protein sequences and premature termination of translation, leading to the likelihood of generating a functional protein is negligible which is the primary mechanism of disease development [26].

The proband of Family 2 carried the c.1428 + 2 T > C variant, located at the 5 ^′^ splice donor site of intron 11, where the second base (thymine) was replaced by cytosine. The nucleotides directly adjacent to the intron–exon junctions within the splice region are highly conserved. According to the canonical GT‐AG rule, the 5 ^′^ (donor site) and 3 ^′^ (acceptor site) bases of introns are almost always GT and AG, respectively. In mammalian genomes, the probability of splice sites conforming to the canonical GT‐AG combination is as high as 98.71% [27]. Therefore, splice acceptor site variants occurring in this conserved region are highly likely to be pathogenic. Minigene assay and Western blot results provided strong evidence for the pathogenicity of c.1428 + 2 T > C, confirming that this novel variant disrupts splice acceptor function, leading to complete skipping of exon 11. Abnormal splicing resulted in the deletion of 39 amino acids (p.Lys438_Gln476del), disrupting the EC domain of the *ENG* protein, which is critical for ligand binding and receptor complex formation (Figure 9). The loss of glycosylation at Asn444 and Asn465 may affect protein stability and trafficking, as evidenced by the accumulation of nonglycosylated forms in MUT cells. Thus, we conclude that this novel splice‐site variant is definitively pathogenic.

In conclusion, through whole‐exome sequencing, we identified variants in the *ENG* gene (c.613del, c.1428 + 2 T > C, c.1498dup, and c.322del) in four families. The pathogenicity of the novel heterozygous intronic variant c.1428 + 2 T > C in the *ENG* gene of Family 2 was verified using an in vitro minigene assay and Western blot analysis. Abnormalities in intronic splicing sites are crucial for predicting splicing variants, which in this case led to aberrant *ENG* mRNA splicing, resulting in the skipping of exon 11 (c.1312_1428del) and the production of a shorter protein with deletion of the predicted glycosylation motifs (p.Lys438_Gln476del). The mutations in Families 1, 3, and 4 (c.613del, c.1498dup, and c.322del) are all frameshift mutations, causing premature termination of translation. Furthermore, our study expands the spectrum of *ENG* variants and highlights the significant phenotypic heterogeneity and genetic complexity associated with *ENG* gene variants in HHT1. These findings not only enhance our understanding of the molecular pathogenesis of HHT but also remind clinicians to consider the possibility of concurrent HHT in patients with PAVMs and emphasize the importance of early genetic testing and personalized therapeutic interventions.

## Author Contributions

Yujing Gong and Tingmin Zhou have contributed to the work equally and should be regarded as co‐first authors.

## Funding

This study was supported by the National Natural Science Foundation of China (10.13039/501100001809) (82171701).

## Conflicts of Interest

The authors declare no conflicts of interest.

## Data Availability

The data that support the findings of this study are available on request from the corresponding authors. The data are not publicly available due to privacy or ethical restrictions.
